# 2,2′-(*p*-Phenyl­ene)bis­(1,4,5,6-tetra­hydro­pyrimidinium) bis­[dicyanidoargentate(I)]

**DOI:** 10.1107/S1600536808015791

**Published:** 2008-06-07

**Authors:** Zhi-Yu Jiang, Hua-Ze Dong, Gong Zhang, Lin Cheng

**Affiliations:** a Department of Chemistry and Chemical Engineering, Southeast University, Nanjing, People’s Republic of China; b Department of Chemistry and Chemical Engineering, State Key Laboratory of Coordination Chemistry, Nanjing University, Nanjing, People’s Republic of China

## Abstract

The asymmetric unit of the title compound, (C_14_H_20_N_4_)[Ag(CN)_2_]_2_, contains one-half of a centrosymmetric 2,2′-(*p*-phenyl­ene)bis­(1,4,5,6-tetra­hydro­pyrimidinium) (H_2_btb) cation and one [Ag(CN)_2_]^−^ anion. In the anions, the Ag^I^ atoms adopt near linear coordination modes with the two attached cyanide groups [C—Ag—C = 173.3 (2)°]. In the crystal structure, each H_2_btb cation links four [Ag(CN)_2_]^−^ anions via N—H⋯N hydrogen bonds into a one-dimensional ribbon.

## Related literature

For related structures, see: Braga *et al.* (2000 ▶); Felix *et al.* (1998 ▶). For related literature, see: Burchell *et al.* (2004 ▶); Holliday & Mirkin (2001 ▶).
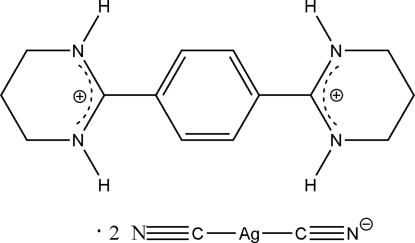


## Experimental

### 

#### Crystal data

(C_14_H_20_N_4_)[Ag(CN)_2_]_2_
*M* *_r_* = 564.16
Triclinic, 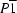

*a* = 6.6930 (9) Å
*b* = 7.276 (1) Å
*c* = 11.4982 (15) Å
α = 89.963 (2)°
β = 87.318 (2)°
γ = 68.066 (2)°
*V* = 518.76 (12) Å^3^
*Z* = 1
Mo *K*α radiation
μ = 1.91 mm^−1^
*T* = 273 (2) K
0.20 × 0.18 × 0.15 mm

#### Data collection

Bruker SMART CCD area-detector diffractometer
Absorption correction: multi-scan (*SADABS*; Bruker, 1998 ▶) *T*_min_ = 0.702, *T*_max_ = 0.763
4040 measured reflections
2015 independent reflections
1769 reflections with *I* > 2σ(*I*)
*R*_int_ = 0.015

#### Refinement

*R*[*F* ^2^ > 2σ(*F* ^2^)] = 0.037
*wR*(*F* ^2^) = 0.093
*S* = 1.09
2015 reflections
135 parameters
H atoms treated by a mixture of independent and constrained refinement
Δρ_max_ = 1.07 e Å^−3^
Δρ_min_ = −0.33 e Å^−3^

### 

Data collection: *SMART* (Bruker, 1998 ▶); cell refinement: *SMART*; data reduction: *SAINT-Plus* (Bruker, 1998 ▶); program(s) used to solve structure: *SHELXS97* (Sheldrick, 2008 ▶); program(s) used to refine structure: *SHELXL97* (Sheldrick, 2008 ▶); molecular graphics: *SHELXTL* (Sheldrick, 2008 ▶); software used to prepare material for publication: *SHELXTL*.

## Supplementary Material

Crystal structure: contains datablocks I, global. DOI: 10.1107/S1600536808015791/bt2718sup1.cif

Structure factors: contains datablocks I. DOI: 10.1107/S1600536808015791/bt2718Isup2.hkl

Additional supplementary materials:  crystallographic information; 3D view; checkCIF report

## Figures and Tables

**Table 1:** Hydrogen-bond geometry (Å, °)

*D*—H⋯*A*	*D*—H	H⋯*A*	*D*⋯*A*	*D*—H⋯*A*
N1—H1*C*⋯N4^i^	0.78 (4)	2.13 (4)	2.903 (4)	175 (4)
N2—H2*C*⋯N3	0.79 (4)	2.13 (4)	2.905 (5)	168 (3)

Symmetry code: (i) 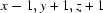.

**Fig. 1.**
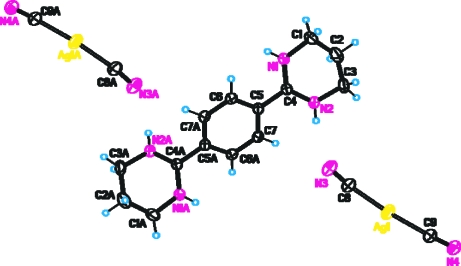
Perspective view of the title compound with 30% displacement ellipsoids. H atoms bonded to C atoms have been omitted for clarity. [Symmetry code (A): 3-x, -y, 1-z.]

**Fig. 2.**
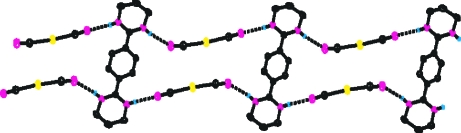
The hydrogen-bonding pattern of the title compound.

## supplementary crystallographic information

### Comment

Supramolecular chemistry has been a rapidly growing field concerning with the construction of supramolecular assemblies held together by non-classical chemical interactions in addition to covalent bonds (Holliday & Mirkin, 2001). A variety of weak forces, such as hydrogen bond, π–π stacking, and metal–ligand coordination, have been extensively used in this field (Burchell *et al.*, 2004). Within the various types of organic ligands utilized in assembly of supramolecular structures, tetrahydropyrimidines have attracted considerable interest for their versatile coordination mode with the protonated or deprotonated moiety and potential to form supramolecular aggregates through hydrogen bonding (Braga *et al.*, 2000; Felix *et al.*, 1998).

Herein, we report the crystal structure of the title compound, (C_14_H_20_N_4_).2(C_2_AgN_2_), based on a tetrahydropyrimidine ligand–1,4-bis(1,4,5,6-tetrahydropyrimidin-2-yl)benzene. The asymmetric unit of the title compound, (C_14_H_20_N_4_).2(C_2_AgN_2_), contains half a H_2_btb cation (btb = 1,4-bis(1,4,5,6-tetrahydropyrimidin-2-yl)benzene) and one Ag(CN)_2_ anion. In the compound, each H_2_btb cation links four Ag(CN)_2_ anions by the N—H···N hydrogen bonds into an one-dimensional ribbon. Meanwhile, each pair of adjacent H_2_btb cations are hydrogen-bonded by two parallel Ag(CN)_2_ anions. The hydrogen-bonding distances are 2.904 (5) and 2.905 (6) Å. In one chain, the shortest Ag···Ag distance is 4.218 (2) Å. The distance of adjacent H_2_btb cations seperated by Ag(CN)_2_ anions is 13.655 (3) Å.

### Experimental

A mixture of btb (0.024 g, 0.1 mmol), k[Ag(CN)_2_] (0.010 g, 0.05 mmol), and water (8 ml) was stirred for 1 h at room temperature, and then filtered. The filtrate was allowed to evaporate slowly at room temperature. After 3 weeks, colorless block crystals were obtained in 60% yield (0.034 g) based on btb.

### Refinement

H atoms bonded to N atoms were located in a difference map and they were freely refined. Other H atoms were positioned geometrically and refined using a riding model with C—H = 0.97 Å and with *U*_iso_(H) = 1.2*U*_eq_(C).

### Crystal data

**Table:**

(C_14_H_20_N_4_)[Ag(CN)_2_]_2_	*Z* = 1
*M**_r_* = 564.16	*F*_000_ = 278
Triclinic, *P*1	*D*_x_ = 1.806 Mg m^−^^3^
Hall symbol: -P 1	Mo *K*α radiation λ = 0.71073 Å
*a* = 6.6930 (9) Å	Cell parameters from 783 reflections
*b* = 7.2760 (10) Å	θ = 2.5–28.0º
*c* = 11.4982 (15) Å	µ = 1.91 mm^−^^1^
α = 89.963 (2)º	*T* = 273 (2) K
β = 87.318 (2)º	Block, colourless
γ = 68.066 (2)º	0.20 × 0.18 × 0.15 mm
*V* = 518.76 (12) Å^3^	

### Data collection

**Table:**

Bruker SMART CCD area-detector diffractometer	2015 independent reflections
Radiation source: fine-focus sealed tube	1769 reflections with *I* > 2σ(*I*)
Monochromator: graphite	*R*_int_ = 0.015
*T* = 273(2) K	θ_max_ = 26.0º
φ and ω scans	θ_min_ = 3.0º
Absorption correction: multi-scan(SADABS; Bruker, 1998)	*h* = −8→7
*T*_min_ = 0.702, *T*_max_ = 0.763	*k* = −8→8
4040 measured reflections	*l* = −14→14

### Refinement

**Table:**

Refinement on *F*^2^	Secondary atom site location: difference Fourier map
Least-squares matrix: full	Hydrogen site location: inferred from neighbouring sites
*R*[*F*^2^ > 2σ(*F*^2^)] = 0.037	H atoms treated by a mixture of independent and constrained refinement
*wR*(*F*^2^) = 0.093	*w* = 1/[σ^2^(*F*_o_^2^) + (0.0523*P*)^2^ + 0.1388*P*] where *P* = (*F*_o_^2^ + 2*F*_c_^2^)/3
*S* = 1.09	(Δ/σ)_max_ < 0.001
2015 reflections	Δρ_max_ = 1.07 e Å^−^^3^
135 parameters	Δρ_min_ = −0.33 e Å^−^^3^
Primary atom site location: structure-invariant direct methods	Extinction correction: none

### Special details

**Table:**

Geometry. All e.s.d.'s (except the e.s.d. in the dihedral angle between two l.s. planes) are estimated using the full covariance matrix. The cell e.s.d.'s are taken into account individually in the estimation of e.s.d.'s in distances, angles and torsion angles; correlations between e.s.d.'s in cell parameters are only used when they are defined by crystal symmetry. An approximate (isotropic) treatment of cell e.s.d.'s is used for estimating e.s.d.'s involving l.s. planes.
Refinement. Refinement of *F*^2^ against ALL reflections. The weighted *R*-factor *wR* and goodness of fit *S* are based on *F*^2^, conventional *R*-factors *R* are based on *F*, with *F* set to zero for negative *F*^2^. The threshold expression of *F*^2^ > σ(*F*^2^) is used only for calculating *R*-factors(gt) *etc*. and is not relevant to the choice of reflections for refinement. *R*-factors based on *F*^2^ are statistically about twice as large as those based on *F*, and *R*- factors based on ALL data will be even larger.

### Fractional atomic coordinates and isotropic or equivalent isotropic displacement parameters (Å^2^)

**Table:**

	*x*	*y*	*z*	*U*_iso_*/*U*_eq_	
Ag1	1.75049 (5)	−0.46673 (5)	−0.11338 (2)	0.06556 (17)	
C1	0.7736 (6)	0.2925 (7)	0.3110 (3)	0.0600 (10)	
H1A	0.7672	0.4129	0.2722	0.072*	
H1B	0.6401	0.3208	0.3572	0.072*	
C2	0.8010 (7)	0.1333 (8)	0.2228 (4)	0.0702 (12)	
H2A	0.6862	0.1799	0.1688	0.084*	
H2B	0.7908	0.0185	0.2617	0.084*	
C3	1.0154 (6)	0.0746 (6)	0.1567 (3)	0.0589 (10)	
H3A	1.0439	−0.0470	0.1127	0.071*	
H3B	1.0125	0.1773	0.1023	0.071*	
C4	1.1503 (5)	0.1093 (5)	0.3465 (3)	0.0385 (7)	
C5	1.3309 (5)	0.0529 (5)	0.4257 (3)	0.0370 (6)	
C6	1.3411 (5)	0.1879 (5)	0.5078 (3)	0.0416 (7)	
H6A	1.2346	0.3145	0.5128	0.050*	
C7	1.4921 (5)	−0.1357 (5)	0.4178 (3)	0.0432 (7)	
H7A	1.4869	−0.2265	0.3623	0.052*	
C8	1.6530 (7)	−0.3227 (7)	0.0448 (4)	0.0652 (11)	
C9	1.8252 (6)	−0.5801 (6)	−0.2799 (3)	0.0536 (9)	
N1	0.9572 (4)	0.2250 (5)	0.3863 (3)	0.0459 (7)	
H1C	0.934 (5)	0.254 (5)	0.452 (3)	0.040 (9)*	
N2	1.1869 (5)	0.0453 (5)	0.2384 (2)	0.0456 (7)	
H2C	1.303 (6)	−0.023 (5)	0.214 (3)	0.036 (9)*	
N3	1.5816 (7)	−0.2314 (7)	0.1250 (3)	0.0828 (12)	
N4	1.8570 (5)	−0.6394 (5)	−0.3728 (3)	0.0616 (9)	

### Atomic displacement parameters (Å^2^)

**Table:**

	*U*^11^	*U*^22^	*U*^33^	*U*^12^	*U*^13^	*U*^23^
Ag1	0.0783 (3)	0.0661 (2)	0.0430 (2)	−0.01739 (17)	0.00644 (14)	−0.01324 (14)
C1	0.0390 (18)	0.078 (3)	0.052 (2)	−0.0090 (17)	−0.0110 (16)	0.0055 (19)
C2	0.059 (2)	0.084 (3)	0.067 (3)	−0.024 (2)	−0.028 (2)	0.005 (2)
C3	0.067 (2)	0.068 (2)	0.0389 (19)	−0.0198 (19)	−0.0212 (17)	−0.0030 (17)
C4	0.0378 (16)	0.0433 (17)	0.0334 (15)	−0.0137 (13)	−0.0051 (12)	0.0008 (13)
C5	0.0361 (15)	0.0434 (16)	0.0314 (15)	−0.0144 (13)	−0.0035 (12)	−0.0011 (12)
C6	0.0396 (16)	0.0402 (16)	0.0383 (17)	−0.0071 (13)	−0.0021 (13)	−0.0050 (13)
C7	0.0440 (17)	0.0429 (17)	0.0391 (17)	−0.0119 (14)	−0.0053 (13)	−0.0119 (13)
C8	0.064 (2)	0.074 (3)	0.047 (2)	−0.015 (2)	0.0055 (18)	−0.010 (2)
C9	0.055 (2)	0.056 (2)	0.047 (2)	−0.0178 (16)	0.0016 (16)	−0.0061 (17)
N1	0.0383 (14)	0.0607 (18)	0.0327 (15)	−0.0112 (12)	−0.0053 (11)	−0.0030 (13)
N2	0.0430 (15)	0.0557 (17)	0.0335 (14)	−0.0126 (13)	−0.0065 (12)	−0.0061 (12)
N3	0.085 (3)	0.097 (3)	0.052 (2)	−0.020 (2)	0.0103 (19)	−0.023 (2)
N4	0.0624 (19)	0.071 (2)	0.048 (2)	−0.0220 (17)	0.0042 (15)	−0.0109 (16)

### Geometric parameters (Å, °)

**Table:**

Ag1—C9	2.050 (4)	C4—N1	1.312 (4)
Ag1—C8	2.052 (4)	C4—C5	1.481 (4)
C1—N1	1.466 (4)	C5—C6	1.385 (4)
C1—C2	1.493 (7)	C5—C7	1.393 (4)
C1—H1A	0.9700	C6—C7^i^	1.376 (4)
C1—H1B	0.9700	C6—H6A	0.9300
C2—C3	1.503 (6)	C7—C6^i^	1.376 (4)
C2—H2A	0.9700	C7—H7A	0.9300
C2—H2B	0.9700	C8—N3	1.115 (6)
C3—N2	1.471 (4)	C9—N4	1.133 (5)
C3—H3A	0.9700	N1—H1C	0.78 (4)
C3—H3B	0.9700	N2—H2C	0.79 (4)
C4—N2	1.307 (4)		
			
C9—Ag1—C8	173.24 (15)	N2—C4—C5	119.5 (3)
N1—C1—C2	108.6 (3)	N1—C4—C5	119.0 (3)
N1—C1—H1A	110.0	C6—C5—C7	119.6 (3)
C2—C1—H1A	110.0	C6—C5—C4	120.1 (3)
N1—C1—H1B	110.0	C7—C5—C4	120.3 (3)
C2—C1—H1B	110.0	C7^i^—C6—C5	120.3 (3)
H1A—C1—H1B	108.3	C7^i^—C6—H6A	119.8
C1—C2—C3	111.0 (4)	C5—C6—H6A	119.8
C1—C2—H2A	109.4	C6^i^—C7—C5	120.1 (3)
C3—C2—H2A	109.4	C6^i^—C7—H7A	120.0
C1—C2—H2B	109.4	C5—C7—H7A	120.0
C3—C2—H2B	109.4	N3—C8—Ag1	172.6 (4)
H2A—C2—H2B	108.0	N4—C9—Ag1	177.0 (4)
N2—C3—C2	109.8 (3)	C4—N1—C1	121.6 (3)
N2—C3—H3A	109.7	C4—N1—H1C	121 (3)
C2—C3—H3A	109.7	C1—N1—H1C	117 (3)
N2—C3—H3B	109.7	C4—N2—C3	123.5 (3)
C2—C3—H3B	109.7	C4—N2—H2C	122 (2)
H3A—C3—H3B	108.2	C3—N2—H2C	114 (2)
N2—C4—N1	121.5 (3)		

Symmetry codes: (i) −*x*+3, −*y*, −*z*+1.

### Hydrogen-bond geometry (Å, °)

**Table:**

*D*—H···*A*	*D*—H	H···*A*	*D*···*A*	*D*—H···*A*
N1—H1C···N4^ii^	0.78 (4)	2.13 (4)	2.903 (4)	175 (4)
N2—H2C···N3	0.79 (4)	2.13 (4)	2.905 (5)	168 (3)

Symmetry codes: (ii) *x*−1, *y*+1, *z*+1.
